# Supplementary material for: Direct RNA nanopore sequencing reveals rapid RNA modification changes following glucose stimulation of human pancreatic beta-cell lines
Source: bioRxiv. 2025 Jun 12:2025.06.12.659352. Preprint. [Version 1] doi: 10.1101/2025.06.12.659352 (PMC12190750; doi:10.1101/2025.06.12.659352)
Supplement: 1 [file NIHPP2025.06.12.659352V1-supplement-1.pdf]

bioRxiv preprint doi: https://doi.org/10.1101/2025.06.12.659352; this version posted June 12, 2025. The copyright holder for this preprint (which was not certified by peer review) is the author/funder, who has granted bioRxiv a license to display the preprint in perpetuity. It is made available under aCC-BY 4.0 International license.

Supplemental Tables

bioRxiv preprint doi: https://doi.org/10.1101/2025.06.12.659352; this version posted June 12, 2025. The copyright holder for this preprint (which was not certified by peer review) is the author/funder, who has granted bioRxiv a license to display the preprint in perpetuity. It is made available under aCC-BY 4.0 International license.

S Table 1. Sequencing Summary Statistics. Sample-level summary statistics. Genes and transcripts detected with ≥ 3 reads. Samples denoted by cell line, native or IVT, glucose condition, and replicate number.

Samples	Number of Reads (M)	Percent Aligned Reads	Transcripts Detected	Genes Detected	N50 (kb)	Mean Read Length	Median Read Length
BH1_native_H_r1	19.65	99.96	40254	17372	1.79	1267.05	921
BH1_native_H_r2	20.57	99.94	41016	17578	1.79	1274.18	934
BH1_native_H_r3	18.61	99.94	40258	17447	1.77	1253.05	912
BH1_native_L_r1	21.77	99.94	42129	17747	1.80	1284.09	940
BH1_native_L_r2	21.77	99.94	41050	17517	1.80	1284.09	940
BH1_native_L_r3	21.77	99.94	41964	17615	1.80	1284.09	940
BH3_native_H_r1	17.18	99.94	35605	16013	1.59	1104.12	780
BH3_native_H_r2	15.62	99.97	33826	15537	1.74	1212.03	860
BH3_native_H_r3	15.58	99.97	34055	15626	1.74	1220.17	863
BH3_native_H_r4	18.38	99.96	34966	15827	1.71	1197.75	856
BH3_native_L_r1	16.10	99.92	34925	15652	1.67	1156.41	821
BH3_native_L_r2	15.20	99.97	32247	15116	1.70	1191.79	851
BH3_native_L_r3	9.67	99.96	29300	14430	1.73	1196.87	844
BH3_native_L_r4	19.62	99.96	31957	15069	1.64	1137.10	800
BH1_IVT_H_r1	2.06	99.76	18936	9079	0.475	436.68	366
BH1_IVT_L_r1	2.10	99.72	19415	9226	0.467	427.84	358
BH3_IVT_H_p1	1.46	99.84	17748	8223	0.514	446.79	376
BH3_IVT_H_p2	1.96	99.83	19711	8764	0.519	443.32	360
BH3_IVT_H_r1	4.19	99.66	24744	10201	0.438	384.76	311
BH3_IVT_H_r2	3.12	99.67	21752	9341	0.465	405.82	326
BH3_IVT_H_r3	1.04	99.36	14229	6863	0.413	356.70	289
BH3_IVT_L_r1	3.29	99.77	20306	8818	0.534	468.09	386
BH3_IVT_L_r2	3.46	99.82	21347	9155	0.493	439.39	367
BH3_IVT_L_r3	3.35	99.78	20686	8894	0.477	422.08	347

bioRxiv preprint doi: https://doi.org/10.1101/2025.06.12.659352; this version posted June 12, 2025. The copyright holder for this preprint (which was not certified by peer review) is the author/funder, who has granted bioRxiv a license to display the preprint in perpetuity. It is made available under aCC-BY 4.0 International license.

S Table 2. RNA modification effector DGE and DTE results. Gene or transcript expression changes for known RNA modification writer, reader, and eraser genes. We report the minimum *P*-value and *q*-value from the differential expression results for the gene or any of the corresponding transcripts.

Gene Symbol	Effector Type	Modification	Minimum Gene *P*-value	Minimum Gene *q*-value
ADAR	writer	inosine	0.091573	0.849601
NSUN2	writer	m5C	0.230276	0.808251
NSUN6	writer	m5C	0.839333	0.996113
ALYREF	reader	m5C	0.439041	0.907533
RAD52	reader	m5C	0.254160	0.825605
SRSF2	reader	m5C	0.001604	0.141133
YBX1	reader	m5C	0.019135	0.525202
ALKBH1	eraser	m5C	0.175732	0.937246
TET1	eraser	m5C	0.049190	0.553876
TET2	eraser	m5C	0.737255	0.970146
TET3	eraser	m5C	0.626204	0.955300
METTL14	writer	m6A	0.278299	0.858438
METTL3	writer	m6A	0.029178	0.529414
RBM15	writer	m6A	0.267021	0.828117
RBM15B	writer	m6A	0.011761	0.285843
WTAP	writer	m6A	0.186124	0.941964
HNRNPA2B1	reader	m6A	0.014807	0.487587
HNRNPC	reader	m6A	0.038105	0.681313
SRSF10	reader	m6A	0.056839	0.751188
SRSF3	reader	m6A	0.283413	0.836723
YTHDC1	reader	m6A	0.032434	0.456420
YTHDC2	reader	m6A	0.694808	0.963389
YTHDF1	reader	m6A	0.970352	0.997533
YTHDF3	reader	m6A	0.123122	0.892943
ALKBH5	eraser	m6A	0.132907	0.712594
FTO	eraser	m6A	0.661733	0.959751
YTHDF2	reader	m6A & m5C	0.176247	0.757396
PUS1	writer	pseudouridine	0.060925	0.576212
PUS10	writer	pseudouridine	0.595974	0.986601
PUS3	writer	pseudouridine	0.698064	0.992503
PUS7	writer	pseudouridine	0.235122	0.970057
PUS7L	writer	pseudouridine	0.312016	0.847353
PUSL1	writer	pseudouridine	0.047473	0.529734
RPUSD1	writer	pseudouridine	0.282757	0.988666
RPUSD2	writer	pseudouridine	0.734146	0.969895
RPUSD3	writer	pseudouridine	0.075340	0.619455
RPUSD4	writer	pseudouridine	0.231712	0.808808
TRUB1	writer	pseudouridine	0.543655	0.937439
PFN1	reader	pseudouridine	0.033006	0.461556

bioRxiv preprint doi: https://doi.org/10.1101/2025.06.12.659352; this version posted June 12, 2025. The copyright holder for this preprint (which was not certified by peer review) is the author/funder, who has granted bioRxiv a license to display the preprint in perpetuity. It is made available under aCC-BY 4.0 International license.

Supplemental Figures

bioRxiv preprint doi: https://doi.org/10.1101/2025.06.12.659352; this version posted June 12, 2025. The copyright holder for this preprint (which was not certified by peer review) is the author/funder, who has granted bioRxiv a license to display the preprint in perpetuity. It is made available under aCC-BY 4.0 International license.

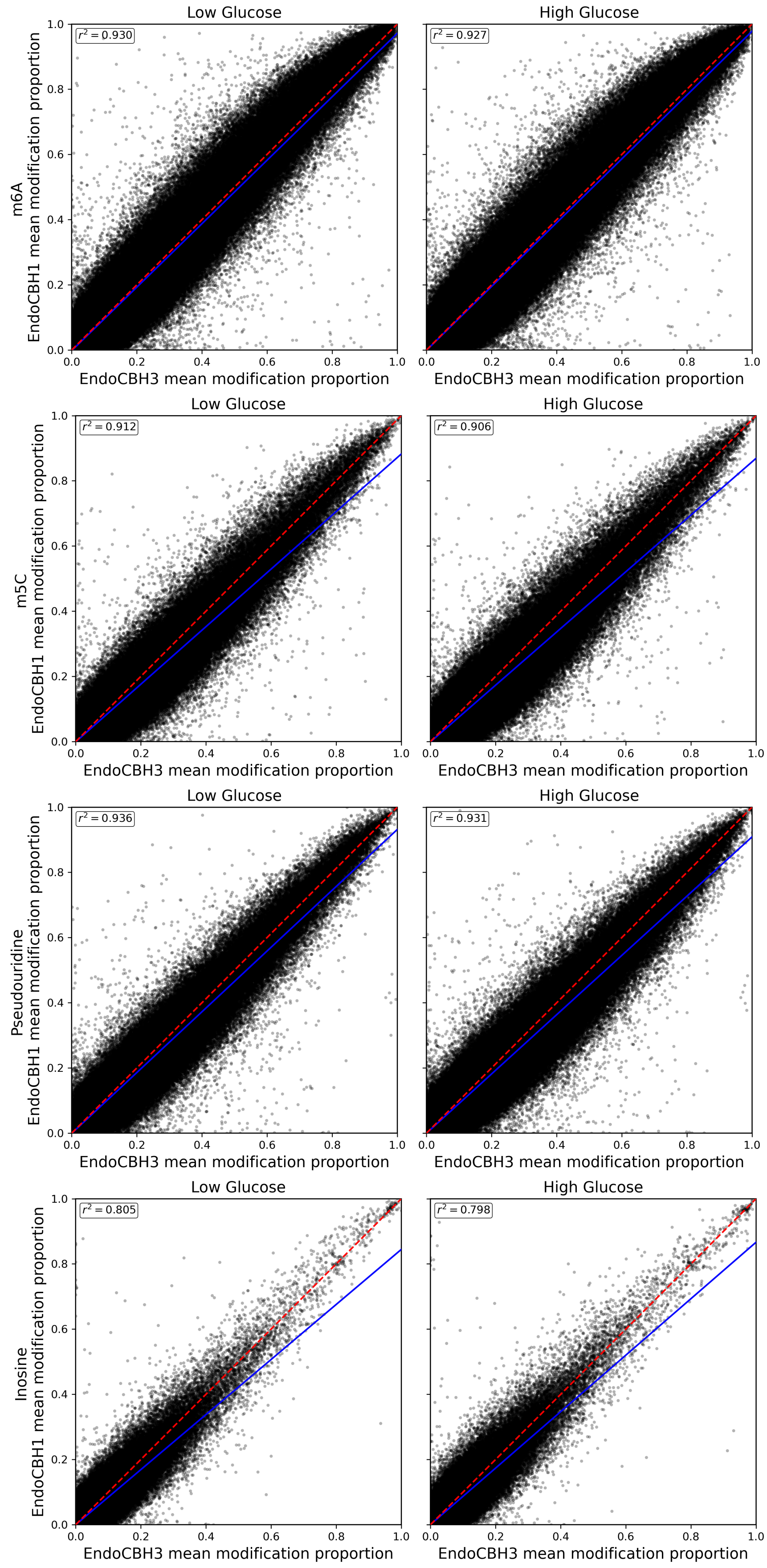

S Fig. 1. Correlation of EndoC-BH1 and EndoC-BH3 modification proportion. Correlation of average RNA modification proportion between EndoC-BH1 (y-axis) and EndoC-BH3 (x-axis) for low and high glucose conditions (facets x-axis) and for the RNA modifications considered (facets y-axis). Dashed red line corresponds to the identity line; the blue line corresponds to the least squares regression line.

bioRxiv preprint doi: https://doi.org/10.1101/2025.06.12.659352; this version posted June 12, 2025. The copyright holder for this preprint (which was not certified by peer review) is the author/funder, who has granted bioRxiv a license to display the preprint in perpetuity. It is made available under aCC-BY 4.0 International license.

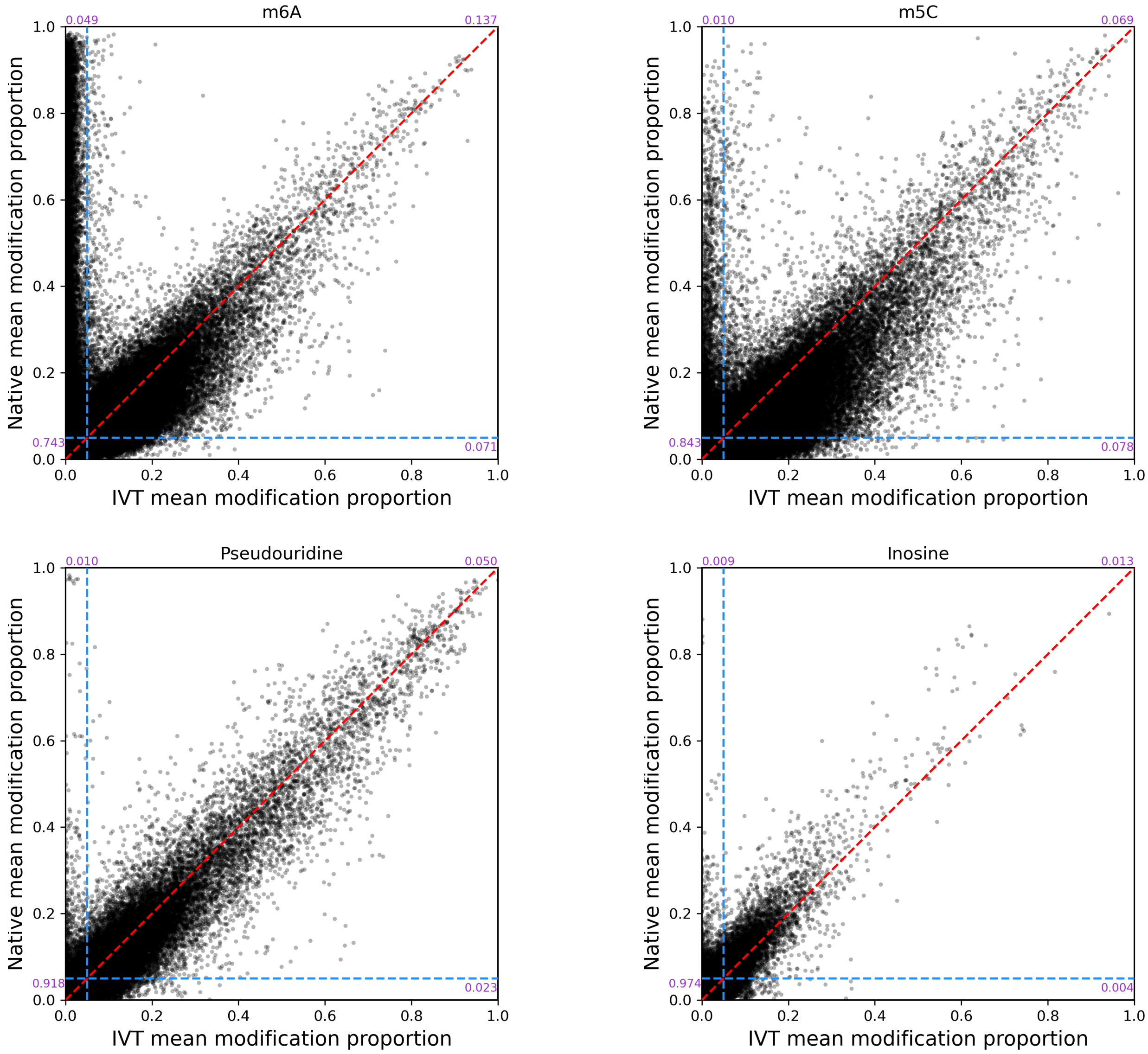

S Fig. 2. Correlation of Native and IVT modification proportion. Correlation of average RNA modification proportion between native (y-axis) and IVT (x-axis) for the RNA modifications considered (facets). Red line corresponds to the identity line; dashed blue lines depict the filtering thresholds. The purple values report the proportion of points within each quadrant defined by the blue, filtering threshold lines.

bioRxiv preprint doi: https://doi.org/10.1101/2025.06.12.659352; this version posted June 12, 2025. The copyright holder for this preprint (which was not certified by peer review) is the author/funder, who has granted bioRxiv a license to display the preprint in perpetuity. It is made available under aCC-BY 4.0 International license.

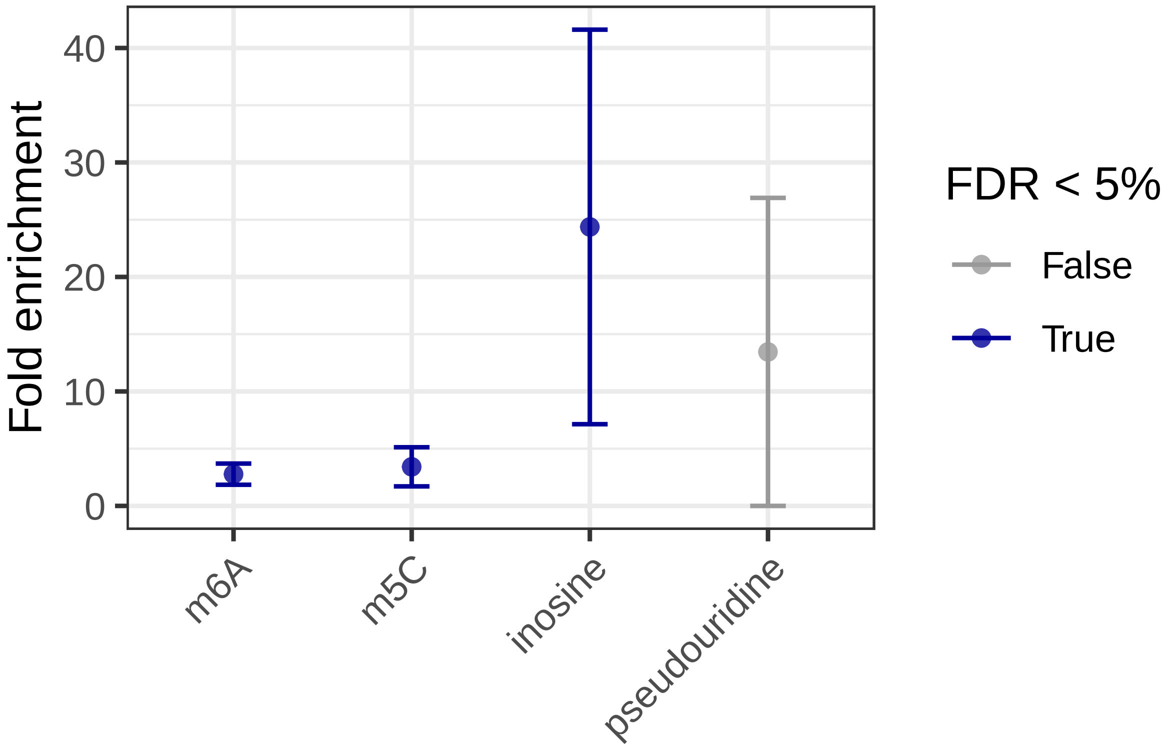

S Fig. 3. Enrichment of known T2D genes. The fold enrichment for T2D genes in the DMSs (y-axis) for the RNA modifications considered (x-axis). Color indicates FDR$< 5\%$.

bioRxiv preprint doi: https://doi.org/10.1101/2025.06.12.659352; this version posted June 12, 2025. The copyright holder for this preprint (which was not certified by peer review) is the author/funder, who has granted bioRxiv a license to display the preprint in perpetuity. It is made available under aCC-BY 4.0 International license.

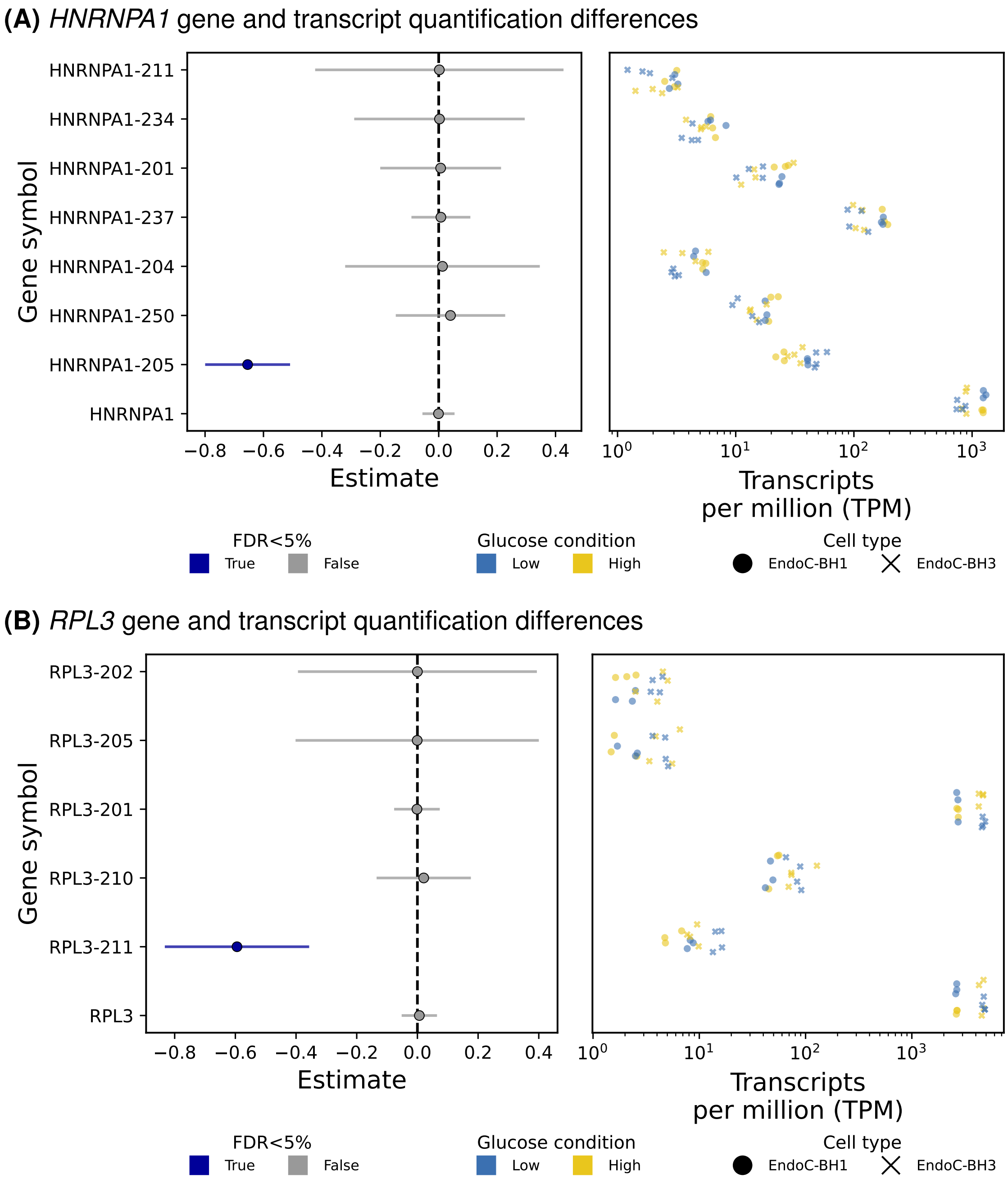

S Fig. 4. Gene and transcript effects. Forest plot (left) and corresponding sample transcripts per million (TPM) (right) for (A) *HNRNPA1* and (B) *RPL3*. The forest plot shows the effect size estimate (x-axis) for each transcript (y-axis). FDR$< 5\%$ shown by blue points and the 95% confidence interval represented by the error lines. The right plot shows the TPM values (x-axis) for the corresponding gene or transcript (y-axis) across samples (points), colored by glucose condition. Point shape distinguishes cell line: EndoC-BH1 (circle) or EndoC-BH3 (X).

bioRxiv preprint doi: https://doi.org/10.1101/2025.06.12.659352; this version posted June 12, 2025. The copyright holder for this preprint (which was not certified by peer review) is the author/funder, who has granted bioRxiv a license to display the preprint in perpetuity. It is made available under aCC-BY 4.0 International license.

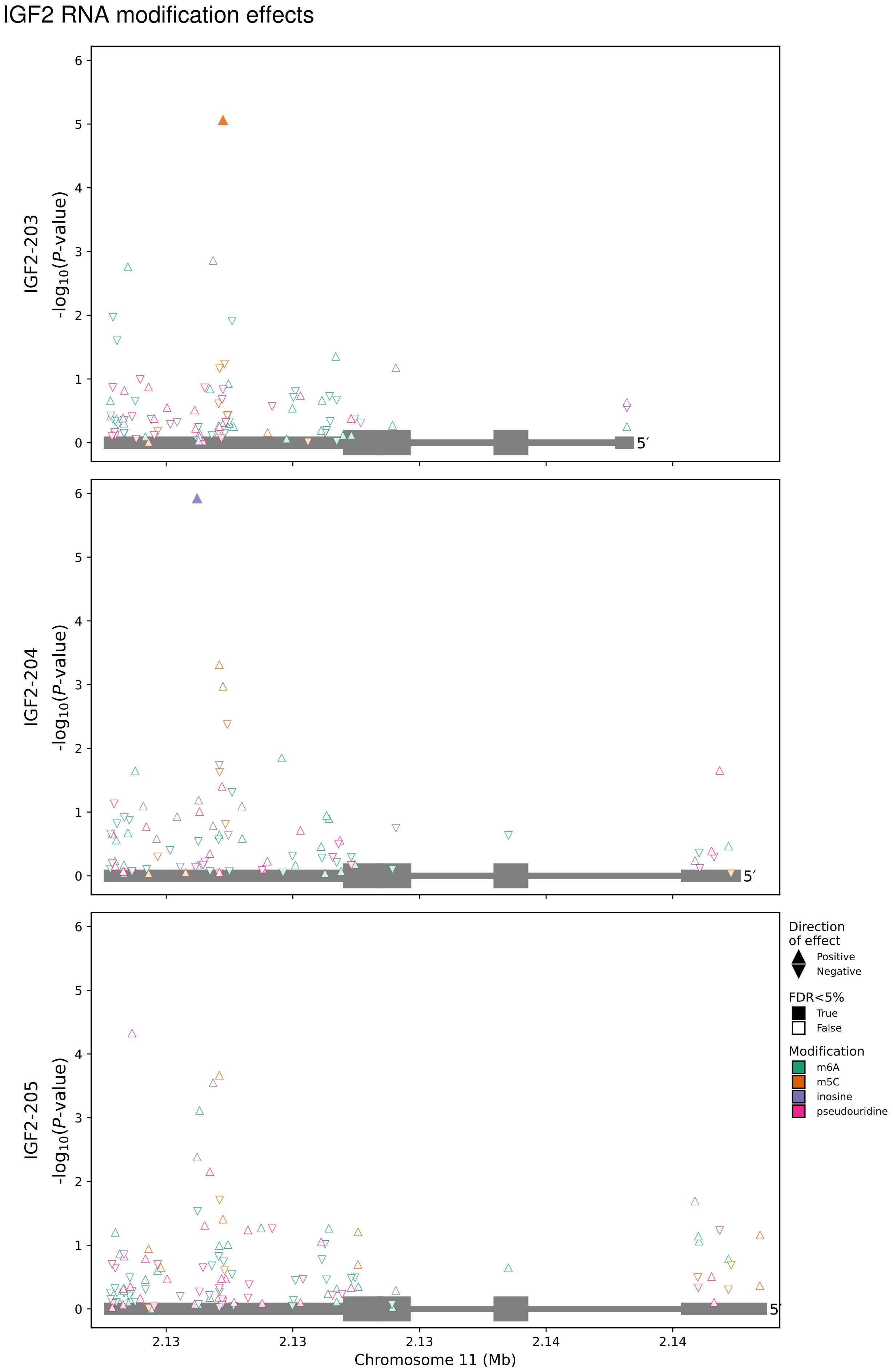

S Fig. 5. ***IGF2*** **RNA modification results at transcript resolution.**

bioRxiv preprint doi: https://doi.org/10.1101/2025.06.12.659352; this version posted June 12, 2025. The copyright holder for this preprint (which was not certified by peer review) is the author/funder, who has granted bioRxiv a license to display the preprint in perpetuity. It is made available under aCC-BY 4.0 International license.

S Fig. 5. *IGF2* RNA modification results at transcript resolution (continued). RNA modification sites considered (points) along with their genomic coordinates (x-axis) in relationship to the detected transcripts (y-axis facets) of *IGF2* (grey boxes) and the differential modification $-log_{10}(P\text{-value})$ (y-axis). Direction of effect indicated by triangle orientation, where an upward triangle indicates a positive effect (i.e., increased proportion of modified counts in the high glucose condition). Colors depict the RNA modification. Fill status indicates FDR$< 5\%$.

bioRxiv preprint doi: https://doi.org/10.1101/2025.06.12.659352; this version posted June 12, 2025. The copyright holder for this preprint (which was not certified by peer review) is the author/funder, who has granted bioRxiv a license to display the preprint in perpetuity. It is made available under aCC-BY 4.0 International license.

GO terms enriched in differential expression results

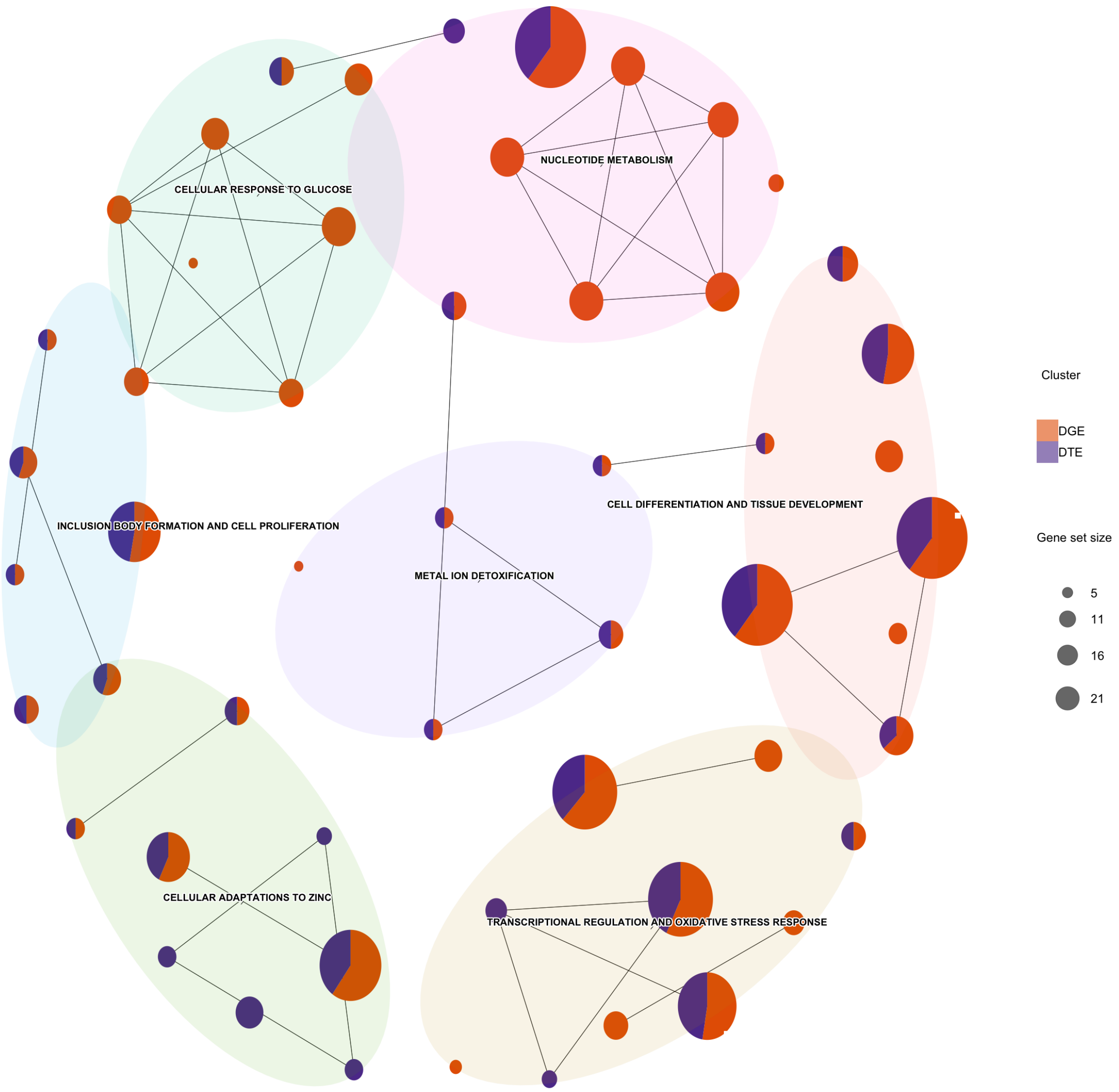

S Fig. 6. GO enrichments for differential gene and transcript expression results Network of GO terms enriched (FDR$< 5\%$) for differential gene and transcript expression. Each node is a GO term with the proportion of genes from DGE (orange) or DTE (purple) represented by the pie charts. The node size is scaled by the number of genes overlapping each GO term. Edges connect semantically similar nodes, which are also clustered based on similarity.
